# War of Ontology Worlds: Mathematics, Computer Code, or Esperanto?

**DOI:** 10.1371/journal.pcbi.1002191

**Published:** 2011-09-29

**Authors:** Andrey Rzhetsky, James A. Evans

**Affiliations:** 1 Department of Medicine, University of Chicago, Chicago, Illinois, United States of America; 2 Department of Human Genetics, University of Chicago, Chicago, Illinois, United States of America; 3 Institute of Genomics and Systems Biology, University of Chicago, Chicago, Illinois, United States of America; 4 Computation Institute, University of Chicago, Chicago, Illinois, United States of America; 5 Department of Sociology, University of Chicago, Chicago, Illinois, United States of America; University of Colorado School of Medicine Denver, United States of America

## Abstract

The use of structured knowledge representations—ontologies and terminologies—has become standard in biomedicine. Definitions of ontologies vary widely, as do the values and philosophies that underlie them. In seeking to make these views explicit, we conducted and summarized interviews with a dozen leading ontologists. Their views clustered into three broad perspectives that we summarize as *mathematics*, *computer code*, and *Esperanto*. Ontology as *mathematics* puts the ultimate premium on rigor and logic, symmetry and consistency of representation across scientific subfields, and the inclusion of only established, non-contradictory knowledge. Ontology as *computer code* focuses on utility and cultivates diversity, fitting ontologies to their purpose. Like computer languages C++, Prolog, and HTML, the *code* perspective holds that diverse applications warrant custom designed ontologies. Ontology as *Esperanto* focuses on facilitating cross-disciplinary communication, knowledge cross-referencing, and computation across datasets from diverse communities. We show how these views align with classical divides in science and suggest how a synthesis of their concerns could strengthen the next generation of biomedical ontologies.

**This is an “Editors' Outlook” article for** ***PLoS Computational Biology***

## Introduction

Historically, ontology was defined as philosophical inquiry into the nature and categories of existence. At the turn of the 20th century, logicians extended and formalized the notion of ontology as a system for describing entities that exist in the world [1], their properties, interrelations, and inferential mechanisms for reasoning about them. In the 1990s, computer scientists reinvigorated and popularized the term by applying it to a wide range of machine-readable knowledge representations. Ontologies could be reused and shared as information schemas [2]. With the rise of scientific databases that are increasingly complex and persistent and require interoperability, ontologies have become enlisted in information technology used by many thousands of specialists worldwide.

In biomedicine today, the term *ontology* means different things to different experts. These meanings range from unordered *terminologies*, to *taxonomies* (terminologies ordered into hierarchical trees), to *formal ontologies* that define object properties and relationships, sometimes including axioms and inference machinery.

An example of a biomedical *terminology* is the American Medical Association's list of Current Procedural Terminology (CPT) codes [3]–[6]. A commonly used *taxonomy* is the International Classification of Diseases (ICD), which organizes disease categories by hierarchical “is-a” relations (e.g., “Breast Cancer *is-a* Malignant Neoplasm”) [7]. Progressively richer *formal ontologies* with multiple types of relations include the Gene Ontology (GO), used to annotate gene products from many model organisms. The GO contains hierarchical “is-a”, “part-of”, and “regulates” relations [8]. Even more involved is the Foundational Model of Anatomy (FMA), which contains a rich set of entity properties and relations that correspond to the networked components of the human body [9], and the BioCyc and MetaCyc ontologies that describe genetic, regulatory, and metabolic cellular pathways of various organisms and enable formal reasoning across those paths [10]. There is disagreement in the community, however, about even these classifications, with some viewing ICD and GO primarily as controlled terminologies with minimal, inconsistent structure.

Ontologies are used for a variety of purposes, from billing patients for medical procedures by a hospital (CPT, ICD) to annotating experimental findings with computer-readable codes for biomedical applications (GO) to reasoning across annotated findings for novel insight (FMA, BioCyc). Biomedical ontologies are often engineered by heterogeneous groups of computer scientists, bench biologists, bedside physicians, programmers, philosophers, and self-identifying ontologists we hereafter collectively refer to as “ontologists.”

Ontologists frequently collaborate on large ontology projects like ICD or GO, but their assumptions about the same ontologies are not universally shared. Publications and conferences about ontologies typically focus on the details of ontology construction and use, but rarely provide a setting for experts to reflect on their understanding of ontologies as knowledge representations. When public reflection does occur, it often escalates to a scuffle of emotionally charged opinion. In seeking to explicate and compare assumptions about ontologies, we collected and recorded views from 14 leading ontologists, selected based on their stature within the ontology community and the diversity of their perspectives. This essay reflects an attempt to summarize the wide range of ontology worldviews revealed through these expert interviews and reflected in ontology projects today. Our summary takes the form of three archetypal views or caricatures that highlight essential differences. While at least two ontologists formulated strong versions of each archetype, others expressed intermediate views. We argue, however, that virtually every current perspective could be represented as a weighted mixture of these three archetypes, much as color visible to the human eye can be expressed as varying intensities of red, blue, and green.

Several characteristics of ontologies were valued widely within the community. For example, all agreed that a good ontology should be logically consistent, structurally acyclic, parsimonious, and elegant. Nevertheless, our informants placed different weight on these virtues and several desired qualities that conflicted with them. Based on these differences, ontology views cohere into three groups that we call *mathematics*, *computer code*, and *Esperanto*.

Table 1 summarizes the primary training and views of the ontologists we interviewed. Many of those interviewed, whose primary training was in linguistics or computer science, are now predominantly working in computational biology or clinical and biomedical informatics.

**Table 1:** Training and Views of Ontologists Interviewed.

Primary Training	#	Mathematics	Computer Code	Esperanto
Computer Science/Artificial Intelligence	3	1+.5+.5	.5+.5	
Linguistics	3		.5+.5	1+.5+.5
Philosophy	1	1		
Clinical and Bioinformatics	4		1+1+1	1
Biology	3	.5	1+.5	1
**Total**	**14**	**3.5**	**6.5**	**4**

## *Mathematics*: Ontology as Formal Theory

The *mathematics* view places a premium on formal consistency in ontologies, with the goal of computational reasoning across them. Some with this view held that a single, unifying ontology covering the whole of biology and medicine is possible to design and desirable to pursue. This unifying ontology need not be complete, and should focus on consensus or uncontested, established knowledge across biomedicine in order to approximate the “underlying reality.” One ontologist holding this position argued that “unless you have a core of terms and relations which is universally valid, however small it might be, then you're always going to have a certain kind of slack in your ontology—the ontology is always going to fall short of being rigorous in the way that arithmetic or even statistics are rigorous.”

This view holds that there is no need to represent uncertainty, hypotheses, or speculations. If probability in representation is combined with a probabilistic form of inference, “then you're going to end up with two successive layers of uncertainty, which will mean that the results will be of quite low value.” First-order logic and computationally tractable subsets of logic are viewed as appropriate tools for conducting inference across rigorous ontologies.

Ontologists voicing the *mathematics* position agreed that despite the current, “chaotic” diversity of ontologies, “every ontology ever built should have the same upper level [ontology], ideally.” In this view, upper level ontologies should precisely define basic categories, such as entities, characteristics, and processes. A few candidates for the role of the upper-level ontologies exist currently (e.g., BFO [11], SUMO [12]–[14], Cyc [15]). Those sharing the *mathematics* view believe that upper-level ontologies will compete for scientific attention until the best emerges and wins out. Computer scientists and the lone philosopher we interviewed were most likely to hold the view of ontologies as *mathematics*.

## *Computer Code*: Ontology as a Custom Code

Another group of ontologists argued that ontologies should be designed specifically for a range of special or general purposes, like the programming languages Prolog and C++ and the mark-up language HTML. One ontologist intimated this metaphor when he revealed that “I view ontologies primarily as software artifacts.” From this perspective, an ontology should primarily aim to serve its function and intended user community, even if small. The specific design choices made in order to achieve the desired utility were viewed as secondary.

This view explicitly opposes the goal of designing a unified ontology for the whole of biomedicine. Instead, the number of ontologies should be equal to or greater than the number of distinct biomedical problems and research needs requiring structured knowledge representation. The most reflective in this cluster described ontologies as “post-modern” traces of conception; “human constructions” assembled to fulfill different needs in distinct social and technical environments rather than “grounded in absolute reality.”

One ontologist voiced a concern common to several when he stated that “overly abstract mathematical ontologies provide a false sense of certainty. They obscure distinctions that might be useful to a particular task, and make unnecessary distinctions.” Practical value should then trump mathematical elegance. These experts considered abstract, upper-level ontologies as so disconnected from the real world that they were dubious about their utility.

Playfully gesturing to Mao Zedong, one ontologist proclaimed “Let a thousand flowers bloom,” suggesting that users should be encouraged to create their own custom ontologies, and that these should be evaluated with regard to usability and efficiency in the context of a specific problem. *Computer code* placed little value on unification, believing that all ontologies can coexist in peace. Medical, clinical, and bioinformatics researchers, as well as the biologists in our sample, most commonly held the view of ontologies as *computer code*—crafted for specific medical or biological projects.

## *Esperanto*: Ontology as Communication Tool

The ontology as *Esperanto* perspective holds that ontologies should facilitate cross-community communication, much like Esperanto, the language constructed by Leyzer Zamenhoff at the end of the 19th century to be easy to learn and politically neutral in the hope of fostering international peace and cooperation. The *Esperanto* position holds that ontologies should cross-link concepts from different domains to allow for the transfer of knowledge and insight between areas, even if imperfectly. This perspective is motivated by the possibility of making data computable over fields, experimental techniques, countries, and time periods.

Researchers holding the *Esperanto* view believe that the goal of a single, unified ontology is unrealistic, even if in an ideal world it might facilitate universal scientific communication. The only practical solution is “a federated interlinkage … a grid or a network of ontologies and vocabularies” made possible primarily through attempts to “invoke concepts that are embedded in another ontology, actually use *that* ontology to describe *that* thing.” Like Esperanto, which borrows most of its vocabulary from common, natural languages, this approach of systematically borrowing terms between ontologies is viewed as essential to create productive overlaps that reduce redundancy and facilitate cross-communication. In this scheme, not every term in every ontology is mapped to another, but the mapping is sufficient to enable researchers to compute across datasets as a whole.

Unlike mathematics, where a single person can construct a novel, consistent system (e.g., Hipparchus alone may have invented the foundations of trigonometry), those espousing the *Esperanto* view believe that to best further biomedical science, ontologies must integrate information widely distributed across research labs and communities. In this view, successful ontology creation requires more than deep domain knowledge and design precision. It also requires diplomatic social activity to coordinate between scientists and fields. An ontology is most useful if it not only helps users perform their work, but also facilitates continuing communication and commerce with the rest of the scientific world. Otherwise it is isolating, and those who use it will neither benefit from nor contribute to advances made elsewhere. Among those interviewed, researchers with linguistics training most frequently held the view of ontologies as *Esperanto*—facilitating not only scientific clarity but also communication.

## How These Groups View One Another

These three ontology perspectives respond directly to one another. In several cases, ontologists drew contrasts explicitly, but in some cases we infer likely differences. On the one hand, ontology as *mathematics* suggests that *computer code* and *Esperanto* approaches are messy and inconsistent, even “silly and childish.” From this perspective, *Esperanto* and *computer code* ontologies are inefficient to improve because they lack a clear means of evaluation like logical consistency. One can rarely reason over an ontology produced from these other approaches without using probability to allow for contradiction and error. On the other hand, ontology as *computer code* and *Esperanto* view the *mathematics* approach as utopian, of little practical use, and even potentially sinister: “one mother ontology to serve all purposes and in the darkness bind them.” Specifically, the *computer code* approach sees *mathematics* ontologies as incomplete and unrepresentative of relevant knowledge in an area, and hence unproductive. *Mathematics* ontologies come off as rigid and artificial to domain experts.

The *Esperanto* approach views the *computer code* zeitgeist as eclectic “chaos,” multiplying unnecessary redundancy, and failing to exploit natural opportunities to link knowledge across areas. The *mathematics* approach views *Esperanto* efforts to integrate domain-specific ontologies as compromising half-measures that abandon the potential strength of unification.

## Parallel Divisions

Reminiscent contrasts have animated fierce debates elsewhere in the history of science. In 17th century Europe, the mechanical philosophers, including Descartes, Hobbes, and Spinoza, favored a systemic, logico-deductive approach to science committed to certain truth. This differed from the experimental philosophers, including Bacon, Boyle, and the fledgling Royal Society, that favored experiments and the establishment of a looser, probabilistic notion of truth surrounding the social establishment of “facts” [16]. This also parallels the 1980s fight between “Neats” and “Scruffies” in the Artificial Intelligence (AI) community [17].

Mechanical philosophers and Neats are close to the *mathematics* group in the ontology community, seeking provable solutions—although logical consistency is typically sufficient to satisfy many in the *mathematics* ontology community. Experimental philosophers and Scruffies are closest to the *computer code* group: they rely on heuristics and the metaphor of probability rather than certainty, claiming that a collection of useful, heterogeneous methods is enough [18].

No direct analog to the *Esperanto* group exists in AI, but scientific communication projects like review journals have long attempted to facilitate knowledge transfer between domains. Novel challenges have arisen from rapid growth in the number of biomedical scientists and subcommunities over the past half century. Counteracting this trend, the informatics revolution of the past 20 years has created novel opportunities to link information across these domains. With the rise of the Internet and computing power, natural language processing (NLP) methods have increasingly enabled researchers to extract information from older articles and books, which makes it available for computational modeling. While this new source of old information enables a much richer view of the ontologies underlying scientific discourse, it poses challenges and suggests new opportunities for how to construct, evaluate, and use ontologies to further biomedical advances.

## Ontology Challenges Posed by Text Mining

First, multiple levels of representational granularity coexist across a scientific corpora and often in a single text. For example, a protein methylation event occurring within a human cell may appear in a molecular biology article as a binary relation between an enzyme and the substrate protein (e.g., “PRMT5 methylates histones H3 and H4”). In a chemical article, methylation is more likely to be described as a multistage process involving additional molecules such as the methyl group donor and transient complexes. If we extract information from text we cannot commit to a single level of representation for a phenomenon if we intend our information to retain the fidelity it possessed in its source.

Second, diversity and disagreement persist within scientific communities—and sometimes even scientists—for long periods and sometimes indefinitely [19]. If we attempt to extract information from text without arbitrary censorship, disagreement must be retained.

Third, objects described in ontologies change in time, so their mentions in text may refer to a spectrum of objects rather than a single one. For example, the Aral Sea, once the fourth largest lake in the world, was reduced to 10% of its original size in just a few years as a result of Soviet irrigation projects; its contour changed dramatically, daily. Even astronomical objects are not immutable: Earth's perspective on the Big Dipper will change radically in the coming 100,000 years.

Fourth, theories and their symbols change in time. This is not a problem for ontologies that eschew representation of uncertain theories. It becomes a problem, however, if we want to represent the current state of scientific knowledge. In cell biology research, when tubulin, the globular protein involved in microtubule construction, was discovered, “tubulin” pointed unambiguously to a unique gene and its product. Within the subsequent decade, many other tubulins (α-tubulin, β-tubulin, etc.) were discovered such that “tubulin” now refers to the entire family. Claims about tubulin from the early period become ambiguous with respect to a later ontology.

These challenges suggest a new virtue, most consistent with the *Esperanto* perspective: representativeness [20]. Insofar as ontologies are employed not only to index biomedical knowledge, but to discover it, they must maintain inconsistent biomedical claims, just as research scientists attempt to do. Inconsistencies should not be ignored, as they point to theoretical weaknesses and opportunities.

In conclusion, we suggest the importance of attending to all three ontology perspectives. Mechanical and experimental philosophers, and Neats and Scruffies advanced science by incorporating the concerns of both. We propose that the usability of an ontology for a particular community and purpose should not be compromised. Additional efforts to maximize an ontology's mathematical rigor, given this usability, however, will improve its reuse and facilitate novel, integrative efforts that enable analysis and discovery across the fields of biomedicine.

**Authors' Biographies**

**Andrey Rzhetsky** is a computational biologist at the University of Chicago. He has worked on mathematical modeling for evolutionary biology, approaches to the analysis of large molecular networks, and massive mining of biomedical literature.

**James Evans** is a sociologist at the University of Chicago whose research focuses on metaknowledge—how social, cultural, and technological institutions shape knowledge and are shaped by it. He is particularly interested in the heuristics by which scientists and their patrons approach research and their consequences for science. Evans also works to develop novel methods to extract, represent, contextualize, and compute over knowledge.
